# Development of Carbon Dioxide Embolism During Partial Amniotic Carbon Dioxide Insufflation for Fetoscopic Laser Photocoagulation: A Case Report

**DOI:** 10.7759/cureus.103759

**Published:** 2026-02-17

**Authors:** Tokimitsu Hibino, Yusuke Okui, Takeshi Murakoshi, Yoshie Toba

**Affiliations:** 1 Department of Anaesthesiology, Seirei Hamamatsu General Hospital, Hamamatsu, JPN; 2 Department of Perinatology, Fetal Diagnosis and Therapy, Seirei Hamamatsu General Hospital, Hamamatsu, JPN

**Keywords:** carbon dioxide, fetoscopy, gas embolism, point-of-care echocardiography, twin-to-twin transfusion syndrome

## Abstract

Partial amniotic carbon dioxide (CO_2_) insufflation during fetoscopic laser photocoagulation (FLP) can improve surgical visibility. However, this procedure poses risks of gas embolism.

A 27-year-old woman underwent FLP for twin-to-twin transfusion syndrome under combined spinal-epidural anesthesia. Owing to turbid amniotic fluid, CO_2_ was manually insufflated. Due to intraoperative respiratory discomfort, the anesthesia was converted to general anesthesia with endotracheal intubation. After a 15° lateral table tilt, the patient developed sudden hypotension and hypoxia. A waterwheel murmur was heard on auscultation. Urgent transthoracic echocardiography confirmed CO_2_ bubbles in the right side of the heart with paradoxical migration to the left atrium through a patent foramen ovale. Immediate gas aspiration and head-down positioning led to rapid recovery. The patient was extubated without neurological deficits.

Intrauterine CO_2_ insufflation can cause systemic gas embolism, particularly with manual injection and postural changes. Early diagnosis using point-of-care ultrasonography is crucial for successful management.

## Introduction

Twin-to-twin transfusion syndrome (TTTS) is a serious complication of monochorionic multiple pregnancies, characterized by unbalanced blood flow through placental vascular anastomoses [1]. Fetoscopic laser photocoagulation (FLP) is the definitive treatment for TTTS, aimed at coagulating these anastomoses to dichorionize the placenta [2,3]. The potential maternal complications of FLP are generally rare [4]. Partial amniotic carbon dioxide (CO₂) insufflation (PACI) may be used to improve endoscopic visibility in cases of turbid amniotic fluid [5,6]. While PACI is an established technique with a known safety profile when intrauterine pressures are controlled, maternal CO₂ embolism is an extremely rare but potentially life-threatening complication that is not commonly reported [7]. Although this technique enhances surgical precision, it introduces the potential risk of gas embolism. In this report, we describe a case of carbon dioxide embolism during FLP with PACI, focusing on the critical role of early clinical suspicion and the utility of immediate transthoracic echocardiography (TTE) in facilitating rapid anesthetic intervention.

## Case presentation

A 27-year-old pregnant woman (height: 158 cm; weight: 55.4 kg) at 22 weeks of gestation was referred to our hospital for the treatment of TTTS. The case was classified as Quintero Stage I, because polyhydramnios was the only abnormal finding. However, the rapid progression of polyhydramnios posed an imminent risk of preterm premature rupture of membranes, necessitating FLP. Combined spinal-epidural anesthesia was performed at the L3/4 interspace using 10 mg of hyperbaric bupivacaine and 10 μg of fentanyl, achieving a sensory block level at the seventh thoracic vertebra.

The surgical entry was performed under continuous transabdominal ultrasound guidance. A trocar (FLP trocar, HAKKO Corporation, Tokyo, Japan; 4 × 5 × 150 mm) was successfully inserted into the uterine cavity on the first attempt. Meticulous care was taken to avoid observable uterine vessels to minimize the risk of hemorrhage and potential gas entry through exposed venous channels. The fetoscopic view became obscured by turbid amniotic fluid 6 min into the procedure, necessitating PACI to restore visualization. Although the trocar was designed for mechanical CO_2_ insufflation, retrograde flow of amniotic fluid prevented the use of a standard mechanical insufflator. Consequently, CO_2_ was manually administered via a syringe. Although not the standard method, this was deemed necessary to safely proceed with FLP and remained within the manufacturer’s intended scope of use. After aspirating 300 mL of amniotic fluid, 200 mL of CO_2_ was injected over 1 min. The surgeon performed the injection slowly, manually monitoring the resistance to avoid excessive intrauterine pressure.

**Figure 1:**
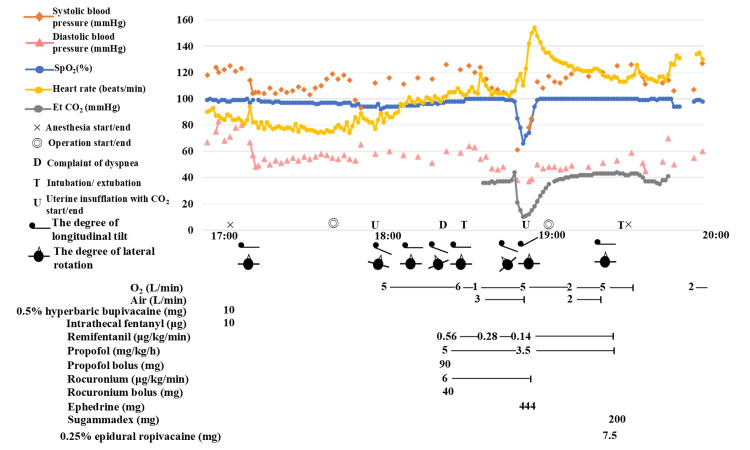
Anesthesia record showing sudden circulatory deterioration during CO2 insufflation CO_2_, carbon dioxide; Et CO_2_, end-tidal carbon dioxide; SpO_2_, peripheral oxygen saturation. Symbols and markers: ×: Start and end of anesthesia; ◎: Start and end of surgery; D: Complaint of dyspnea; T: Tracheal intubation and extubation; U: Start and end of intrauterine CO_2_ insufflation; Side-view silhouette: Longitudinal tilt of the patient; Head-view silhouette: Lateral tilt of the patient A schematic diagram of the patient’s position is shown below the time axis. Immediately after tilting the operating table 15° to the left, sudden decreases in blood pressure, SpO_2_, and end-tidal CO_2_ occurred, followed by compensatory tachycardia. The hemodynamic status improved promptly after cessation of CO_2_ insufflation and subsequent gas aspiration.

Following CO_2_ insufflation, the patient’s SpO_2_ decreased from a baseline of 98% to 93%. This was initially attributed to a combination of intercostal muscle weakness induced by spinal anesthesia and abdominal compression from surgical manipulation. Supplemental oxygen (5 L/min) was initiated, and the patient’s position was adjusted from a 10° reverse Trendelenburg position with 10° left rotation to right rotation, which stabilized the SpO_2_ at 96%-98%. The patient reported dyspnea (SpO_2_: 96% on 5 L/min oxygen) 20 min later. Following a risk-benefit discussion, the procedure was transitioned to general anesthesia. Induction was achieved with propofol (90 mg), remifentanil (0.56 μg/kg/min), and rocuronium (40 mg), followed by uneventful tracheal intubation using a 7.0-mm internal diameter tube. Vital signs remained stable during induction; blood pressure changed from 125/60 to 122/58 mmHg, and the heart rate changed from 88 to 91 beats per minute. Immediately after tilting the operating table 15° to the left to optimize the surgical field, the patient’s hemodynamic status acutely deteriorated: SpO_2_ dropped to 86%, end-tidal CO_2_ decreased precipitously from 44 to 10 mmHg, and blood pressure fell to 61/38 mmHg (Figure 1). The table was immediately returned to a horizontal position and then to the Trendelenburg position. Auscultation revealed a pathognomonic waterwheel murmur at the left sternal border. Emergency TTE confirmed the presence of numerous hyperechoic bubbles in the right heart chambers, with paradoxical migration into the left atrium via a patent foramen ovale (PFO) (Video 1).

**Video 1:** Video of emergency transthoracic echocardiography during the acute event AV, aortic valve; LA, left atrium; LV, left ventricle; RA, right atrium; RV, right ventricle. This video captures the heart shortly after the onset of hemodynamic instability. Numerous highly echoic microbubbles are observed migrating from the RA to the LA and passing through the aortic valve (at 2–3, 8–9, and 14–15 seconds), confirming paradoxical gas embolism. Dilatation of the right heart chamber is also evident.

We immediately requested the cessation of CO_2_ insufflation and aspiration of intrauterine gas. Within 3 min of CO_2_ removal, SpO_2_ increased to 99% and blood pressure stabilized. Repeat TTE showed a marked reduction in hyperechoic bubbles, with minimal new influx into the right atrium (Video 2). Laboratory findings 28 min after the event revealed significantly elevated fibrin degradation products (646.4 μg/mL) (Table 1); however, the criteria for disseminated intravascular coagulation were not met [8]. An arterial blood gas analysis was attempted following the event; however, the sample could not be analyzed due to a measurement error. Consequently, parameters such as pH, PaO_2_, PaCO_2_, and lactate were not obtained.

**Video 2:** Video of transthoracic echocardiography showing the inferior vena cava (IVC) at the end of the surgery CO_2_, carbon dioxide; IVC, inferior vena cava; RA, right atrium. A longitudinal view of the IVC in the epigastric region was obtained after hemodynamic stabilization. The confluence of the hepatic veins and the IVC is visualized. While occasional high-echoic microbubbles are still observed flowing from the caudal side toward the right atrium (RA), their frequency and density are markedly reduced compared to those shown in Video 1. This finding suggests a marked decrease in CO_2_ entry from the uterine cavity into the venous system.

**Table 1:** Temporal changes in coagulation parameters -, not measured; APTT, activated partial thromboplastin time; DIC, disseminated intravascular coagulation; FDP, fibrin and fibrinogen degradation products; INR, international normalized ratio; NA, not available; PT, prothrombin time. Day: Number of days before (−) or after (+) surgery, with Day 0 being the day of surgery. Values in parentheses indicate reference ranges at our institution. Changes in each parameter were described based on the DIC criteria by Wada et al. [9].

	Day -23	Day -10	Crisis during FLP	Day +1	Day +3	Day +12	In DIC
PT (s) (9.6-13.1)	10	9.6	10.2	11	9.3	9.8	Prolongation
PT-INR (0.85-1.10)	0.91	0.87	0.94	1	0.85	0.9	Prolongation
APTT (s) (24-34)	31.5	29.5	34.8	35.5	32.2	27	Biphasic waveform
Fibrinogen (mg/dL) (200-400)	351	470	421	344	472	472	Reduction
D-dimer (μg/mL) (<1.0)	4.3	1.2	86.3	65.2	13.6	1.6	Elevation
FDP (μg/mL) (<5.0)	-	-	646.4	-	-	-	Elevation
Antithrombin(%) (80-130)	-	-	76	-	-	-	Reduction
Platelet count (× 10^9^/L) (158-348)	169	245	120	127	123	299	Reduction

The surgery was aborted. Upon removal of the trocar, no amniotic fluid leakage was observed from the uterine wall, and no suturing was required. Transabdominal ultrasound performed immediately following the procedure confirmed the presence of stable cardiac activity in both fetuses. The patient was extubated after regaining consciousness. Postoperatively, she reported a mild headache that was not exacerbated by positional changes (supine to semi-sitting), suggesting it was a sequela of CO_2_ embolization rather than a post-dural puncture headache. The headache resolved spontaneously within 45 min without neurological deficits. The twins survived and were delivered via cesarean section at 32 weeks with favorable Apgar scores.

This case confirms that high clinical suspicion combined with the immediate application of bedside TTE is pivotal for the diagnosis of CO_2_ embolism. Such rapid detection facilitates the prompt cessation of CO_2_ insufflation, thereby preventing further gas entry and averting catastrophic maternal deterioration.

## Discussion

This case highlights a rare but life-threatening case of CO₂ embolism during PACI, triggered by postural changes. The rapid decline in SpO_2_, end-tidal CO_2_, and blood pressure, combined with a waterwheel murmur, strongly suggested gas embolism. Although arterial blood gas data were unavailable due to a technical error, the diagnosis of CO_2_ embolism was clinically evident. The pathognomonic finding was the precipitous drop in EtCO_2_ from 44 to 10 mmHg, which is a hallmark of increased alveolar dead space caused by pulmonary gas embolism. Furthermore, the rapid hemodynamic recovery within three minutes of ceasing insufflation is highly characteristic of CO_2_ rather than air, given the high solubility of CO_2_ in blood.

Although we had no prior clinical experience with the waterwheel murmur, its distinctive splashing and gurgling quality allowed for immediate recognition upon the first hearing. This underscores the clinical importance of its unique acoustic signature in diagnosing cardiac air embolism. Point-of-care TTE confirmed numerous microbubbles in the right side of the heart and their paradoxical migration to the left atrium through a PFO, likely due to the right atrial pressure exceeding that of the left (Video 1). If the PFO had been closed, systemic CO₂ embolism would likely have been reduced, but the outcome might have differed. First, if the CO₂ inflow had been small, a mild decrease in SpO_2_ and EtCO₂ would likely have been observed. If the CO₂ inflow had been massive, CO₂ would have severely impaired pulmonary circulation. Without intracardiac shunting to the left atrium, the left ventricle would have lost its preload, leading to a rapid decrease in cardiac output and an even more severe circulatory collapse.

**Figure 2:**
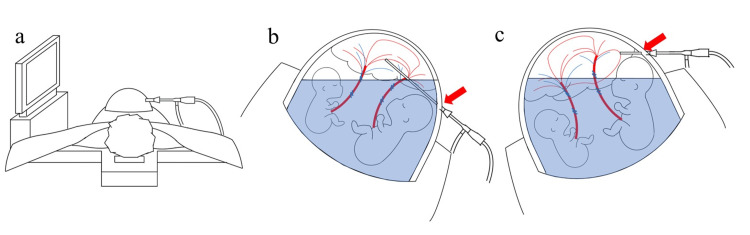
Fetoscopic laser photocoagulation (FLP) setup and conceptual mechanism of CO2 entry Image credits: Created by the authors of this study. CO_2_, carbon dioxide; FLP, fetoscopic laser photocoagulation. a: Surgical setup for FLP. Preoperative evaluation of fetal and placental positions and the distribution of anastomosing vessels is essential for determining the optimal trocar insertion site. b: Schematic of the uterine cavity with the operating table tilted to the right. The maternal abdominal wall is omitted for clarity. The red arrow indicates the trocar insertion site. The amniotic fluid remains above this site, preventing contact between the CO_2_ gas layer and the uterine wall entry point. c: Schematic of the uterine cavity with the operating table tilted to the left. The red arrow indicates the trocar insertion site (now exposed to the gas layer). The shift in the amniotic fluid level exposes the entry point to the CO_2_, facilitating gas entry into the maternal circulation via the injured uterine veins.

The source of CO_2_ was intrauterine insufflation. Regarding the clinical course, CO₂ embolism did not occur during the initial phase with a 10° lateral tilt. We believe that at this angle, the CO₂ gas within the uterine cavity remained separated from the trocar insertion site. However, when the tilt was increased to 15°, the intrauterine gas likely shifted, bringing it into direct contact with the exposed vessels at the insertion site. As illustrated in Figure 2, this contact is considered the critical point that allowed the CO₂ to enter the venous system, subsequently leading to the embolic event. Additionally, CO₂ flowed into the venous system in the reverse Trendelenburg position. Therefore, the exposed vessel was located below the heart, and the CO₂ inflow was not due to a height difference. This suggests that CO₂ inflow can occur even when the exposed vessel is not higher than the heart, and clinicians should be mindful that gas embolism can occur.

Regarding the surgical technique, the trocar was inserted smoothly into the uterus on the first attempt. The operating surgeon noted CO₂ leakage from the uterine wall, which was identified by the gradual loss of the intrauterine gas space and a subsequent decrease in surgical visibility. To maintain an adequate working environment, supplemental CO₂ was manually insufflated as needed. Therefore, even though trocar insertion was smooth, CO₂ was passing through the uterine wall. If this procedure had required multiple attempts, the resulting extensive damage to the uterine wall would likely have exposed the intrauterine venous plexuses, creating a definitive pathway for CO₂ entry. The patient was 22 weeks pregnant. The fundal height at 20 weeks gestation was 20 cm, and at 36 weeks gestation it was 36 cm [10]. Compared to the third trimester, the uterine wall is thicker in the second trimester. Nevertheless, as in this case, CO₂ can pass through the uterine wall, so extra caution is required when performing PACI on patients in the third trimester.

While previous reports suggest that insufflation pressures of 13-22 mmHg are generally safe [11], our use of manual syringe injections, necessitated by a narrow trocar tube, lacked the pressure-limiting safety of an automated insufflator. This likely resulted in transiently high intrauterine pressures, facilitating gas entry into the maternal circulation. Even at lower pressures, CO_2_ has been reported to leak from the uterus into the maternal peritoneal cavity in 27.7% of reported cases [12], demonstrating that the uterine cavity is not always airtight during PACI. Such leakage, combined with postural changes, may further increase the risk of gas being forced into the venous system under suboptimal pressure control.

Although a meta-analysis of 9,403 fetoscopy cases did not reveal that gas embolism was a potential maternal complication of the procedure [13], its possibility should be considered during PACI. Amniotic fluid embolism was a key differential diagnosis in the present case. It is difficult to directly extrapolate what occurs in gases to liquids, but it cannot be ruled out that amniotic fluid may also enter the venous system from exposed blood vessels in the uterine wall. However, our patient’s clinical course lacked the classic triad of amniotic fluid embolism-bronchospasm, profound shock, and rapid-onset disseminated intravascular coagulation. Furthermore, the characteristic echocardiographic findings, i.e., uniform, highly echogenic microbubbles rather than the varying size, shape, and echogenicity typical of amniotic fluid embolism [14,15], confirmed the diagnosis of a CO_2_ embolism. Crucially, the rapid clinical recovery within minutes of gas aspiration and cessation of insufflation further supports CO_2_ as the causative agent, given its high solubility compared to that of air or the persistent nature of amniotic debris.

The paradoxical migration of CO_2_ through the PFO may explain the patient’s postoperative headache, suggesting transient cerebral gas entry. Fortunately, the high solubility of CO_2_ likely prevented permanent neurological deficits. Given that CO_2_ leakage from the uterus can occur even at low pressures [12], any intrauterine gas insufflation carries a latent risk of systemic embolism. In the present case, the cessation of CO_2_ insufflation and subsequent aspiration reduced the amount of CO_2_ passing through the inferior vena cava. Therefore, we propose that point-of-care TTE should be readily available or even proactively conducted and monitored when intrauterine insufflation is performed. Early detection using TTE, combined with immediate clinical suspicion and Trendelenburg positioning, is essential for preventing catastrophic outcomes.

## Conclusions

Intrauterine CO_2_ insufflation during FLP poses a risk for systemic gas embolism, particularly when manual injection or significant postural changes are involved. This rare but severe complication can be rapidly diagnosed using point-of-care TTE and characteristic auscultation findings. Anesthesiologists should maintain a high index of suspicion and prepare for immediate intervention, including gas aspiration and head-down positioning, to ensure maternal and fetal safety during fetal endoscopic procedures.

## References

[1] Ruano R, Rodo C, Peiro JL (2013). Fetoscopic laser ablation of placental anastomoses in twin-twin transfusion syndrome using 'Solomon technique'. Ultrasound Obstet Gynecol.

[2] Slaghekke F, Lopriore E, Lewi L (2014). Fetoscopic laser coagulation of the vascular equator versus selective coagulation for twin-to-twin transfusion syndrome: an open-label randomised controlled trial. Lancet.

[3] Bamberg C, Hecher K (2019). Update on twin-to-twin transfusion syndrome. Best Pract Res Clin Obstet Gynaecol.

[4] Habli M, Bombrys A, Lewis D, Lim FY, Polzin W, Maxwell R, Crombleholme T (2009). Incidence of complications in twin-twin transfusion syndrome after selective fetoscopic laser photocoagulation: a single-center experience. Am J Obstet Gynecol.

[5] Kohl T, Tchatcheva K, Weinbach J, Hering R, Kozlowski P, Stressig R, Gembruch U (2010). Partial amniotic carbon dioxide insufflation (PACI) during minimally invasive fetoscopic surgery: early clinical experience in humans. Surg Endosc.

[6] Sangara RN, Chon AH, Van Speybroeck AL (2021). Fetal blood gases after in utero carbon dioxide insufflation for percutaneous fetoscopic spina bifida repair. Am J Obstet Gynecol MFM.

[7] Kohl T, Reckers J, Strümper D (2004). Amniotic air insufflation during minimally invasive fetoscopic fetal cardiac interventions is safe for the fetal brain in sheep. J Thorac Cardiovasc Surg.

[8] Gando S, Iba T, Eguchi Y (2006). A multicenter, prospective validation of disseminated intravascular coagulation diagnostic criteria for critically ill patients: comparing current criteria. Crit Care Med.

[9] Wada H, Matsumoto T, Yamashita Y (2014). Diagnosis and treatment of disseminated intravascular coagulation (DIC) according to four DIC guidelines. J Intensive Care.

[10] Gasner A, Aatsha PA (2024). Physiology, uterus. StatPearls [Internet].

[11] Kohl T, Tchatcheva K, Berg C, Geipel A, Van de Vondel P, Gembruch U (2007). Partial amniotic carbon dioxide insufflation (PACI) facilitates fetoscopic interventions in complicated monochorionic twin pregnancies. Surg Endosc.

[12] Ziemann M, Fimmers R, Khaleeva A, Schürg R, Weigand MA, Kohl T (2018). Partial amniotic carbon dioxide insufflation (PACI) during minimally invasive fetoscopic interventions on fetuses with spina bifida aperta. Surg Endosc.

[13] Sacco A, Van der Veeken L, Bagshaw E, Ferguson C, Van Mieghem T, David AL, Deprest J (2019). Maternal complications following open and fetoscopic fetal surgery: a systematic review and meta-analysis. Prenat Diagn.

[14] Shaikh N, Alhammad MF, Nahid S, Umm E A, Fatima I, Ummunnisa F, Yaqoub SA (2023). Amniotic fluid embolism causing multiorgan embolisms and reinforces the need for point-of-care ultrasound. Qatar Med J.

[15] Barakat M, Alamami A, Ait Hssain A (2022). Recurrent cardiac arrests due to amniotic fluid embolism. Cureus.

